# Enterotoxin Gene Cluster-Encoded SEI and SE*l*N from *Staphylococcus aureus* Isolates are Crucial for the Induction of Human Blood Cell Proliferation and Pathogenicity in Rabbits

**DOI:** 10.3390/toxins8110314

**Published:** 2016-10-28

**Authors:** Andreas Roetzer, Corina S. Gruener, Guenter Haller, John Beyerly, Nina Model, Martha M. Eibl

**Affiliations:** Biomedizinische ForschungsgmbH, Lazarettgasse 19/2, Vienna A-1090, Austria; andreas.roetzer@biomed-research.at (A.R.); corina.gr@gmx.at (C.S.G.); guenter.haller@biomed-research.at (G.H.); jbeyerly@gmx.at (J.B.); nina.model@biomed-research.at (N.M.)

**Keywords:** *Staphylococcus*, enterotoxin gene cluster, immunogenicity, toxicity

## Abstract

Among the toxin family of bacterial superantigens, the six members of the enterotoxin gene cluster (egc) seem to have unusual characteristics. They are present in the majority of *Staphylococcus aureus* strains, but their role in disease remains uncertain. We assessed secretion levels, immunogenicity, and toxicity of native and recombinant egc proteins. After having developed enzyme-linked immunosorbent assays, we found different quantities of egc proteins secreted by bacterial isolates. Supernatants induced proliferation of human peripheral blood mononuclear cells. However, purified recombinant egc proteins were shown to have differing superantigenicity potentials. Immunization with identical amounts of all members of egc, and the prominent toxic agent SEB, resulted in neutralizing antisera. Two egc proteins, SEI and SE*l*N, were found to play a predominant role within the cluster. Both displayed the highest potential to activate blood cells, and were essential to be neutralized in supernatants. The application of a supernatant of a strain bearing only egc was sufficient for a lethal outcome in a rabbit model. Again, neutralization of SEI and SE*l*N led to the survival of all tested animals. Finally, nanogram amounts of purified rSEI and rSE*l*N led to lethality in vivo, pointing out the importance of both as virulence determinants among egc superantigens.

## 1. Introduction

*Staphylococcus aureus* is known to be one of the most challenging human bacterial pathogens worldwide. Staphylococcal infections can lead to various diseases comprising simple skin abscesses, but also necrotizing pneumonia or fatal sepsis [1,2]. We know of a number of examples of secreted and membrane-associated proteins of *S. aureus*, which are believed to contribute to distinct disease patterns. This arsenal includes hydrolytic enzymes, adhesins, and a large number of toxins, such as superantigens [3,4,5]. Systemic distribution of secreted superantigens was shown to be the cause for a cytokine storm [6]. A large group of T cells are activated through a cascade initiated by the binding of superantigens to MHC class II and the Vβ chains of the T cell receptor. Ultimately, this mechanism leads to toxic shock syndrome (TSS) [7]. 

Genotypes of large cohorts of clinical isolates have been explored extensively [8,9,10,11]. The group of superantigens with the highest prevalence is the so-called enterotoxin gene cluster (egc) including SEG, SEI, SE*l*M, SE*l*N, SE*l*O, and SE*l*U [12]. *Seg* and *sei* were the first genes discovered in this cluster and both were identified to have emetic activity [13]. A few years later, three additional enterotoxin-like open reading frames (*selm*, *seln*, and *selo*) were found in the flanking regions by Jarraud et al., who designated this region egc [12]. Their results also pointed to co-transcription of these genes, leading to the term egc operon. However, alternative start sites could not be ruled out. Among this cluster, two pseudogenes (ϕent-1 and ϕent-2) were described as well. Two years later *selu* was discovered, which resulted from base pair deletions within the two pseudogenes, giving one consecutive open reading frame [14]. Finally, the existence of *selu2* based on different base pair deletions, and *selv* generated by a recombination of *selm* and *sei*, were announced [15].

Reports about the role of the egc operon as a virulence factor remain contradictory. Since this gene cluster can be frequently found in isolates of healthy individuals, as well as in those of patients, their sole presence might not be decisive for their role as a virulence determinant [11,12]. There was no prevalence of egc superantigens among septic shock patients [16]. However, another report stated that isolates from patients with toxic shock syndrome and scarlet fever had nothing else but the egc toxins [17]. A case of a diabetic man was reported, who suffered from necrotizing fasciitis, which, despite antimicrobial therapy, led to the amputation of one leg [18]. The etiological agent was an *S. aureus* strain bearing no other superantigens but the egc cluster. Importantly, Stach et al. have recently described the involvement of egc-encoded proteins in infective endocarditis, proving their specific biological importance in a rabbit model for the first time [19]. 

Clinical studies on antibodies against members of the egc operon are rare [20]. The reason might be their unclarified clinical significance. Findings have demonstrated high genomic frequency in infective staphylococci and the lack of antibodies in exposed individuals. Several possible reasons for this discrepancy have been postulated, e.g., reduced immunogenicity, low expression rates, lack of exposure to relevant immune cells, or even a decrease of responding cells due to exaggerated immune reactions [21,22]. In our study we focused on previously described contemporary staphylococcal strains [23]. Their genetic backgrounds displayed only superantigen toxins belonging to the egc operon. We detected low rates of toxin production. This production of sub-immunogenic concentrations of toxin in the course of infection could explain the lack of antibody production in the majority of individuals. In our studies on immunogenicity, rabbits receiving microgram amounts of the respective recombinant superantigen in parallel displayed a comparable immune response. We confirmed superantigenicity of the individual egc members, and assessed and proved their toxicity in a rabbit model.

## 2. Results

### 2.1. Superantigens of the egc Operon are Produced in Very Low Amounts

In this study, we analyzed immunogenicity and toxicity of members of the egc operon, i.e., SEG, SEI, SE*l*M, SE*l*N, SE*l*O, and SE*l*U. Toxins isolated from *S. aureus* and recombinant egc proteins were explored both in vitro and in a rabbit model. We picked fourteen fresh isolates bearing only egc-associated superantigens out of a cohort of 51 strains (27.4%) obtained from patients from the general hospitals in Vienna and Linz [23]. Four strains were derived from patients with bacteremia (28.5%), the remaining ten colonizing strains were collected from the nasal cavity of healthy individuals (71.5%). As a control, we investigated the characteristics of a colonizing isolate also having *seb* and *seh* beside the egc gene cluster (Table 1). Out of fourteen isolates, seven egc clusters had the identical gene setup, including *seg*, *sei*, *selm*, *seln*, *selo*, and *selu*. Another five strains had the same gene constellation with *seg*, *sei*, *selm*, *seln*, and *selo*. The two remaining egc isolates were lacking *seg*, but had *sei*, *selm*, *seln*, *selo*, and *selu*. The *seb*-positive isolate was harboring all six members of the egc cluster. Comparison of amino acid sequences of the six full-length egc proteins revealed similar percentages of identities (*seg* 97.3%, *sei* 90.5%, *selm* 90.4%, *seln* 85.3%, *selo* 85.8%, *selu* 91.9%).

We explored expression levels of identified members of the egc operon. For transcriptional assessment, strains were cultured in parallel in tryptic soy broth medium at 37 °C. After starting at an identical optical density of 0.02, the onset of transcription became visible after 4 h, and continued to rise slightly until the early stationary growth phase. However, the overall rate of transcription of all members was poor in not exceeding a one-fold induction (Supplementary Materials Figure S1A).

**Figure 1:**
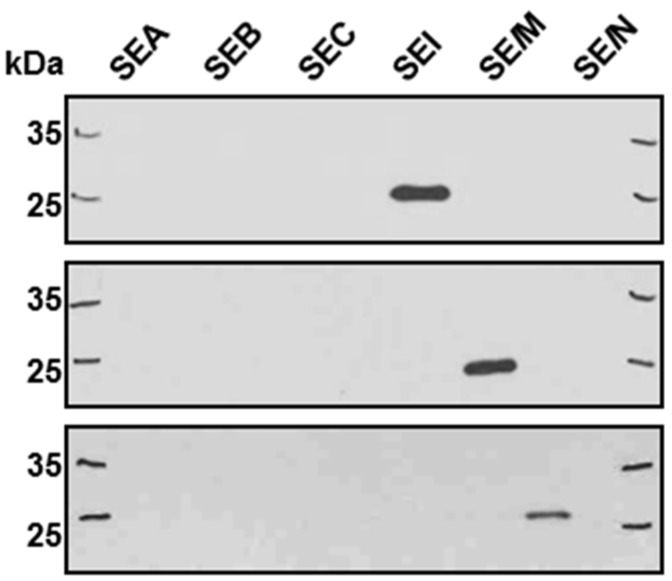
Western blot analysis of specificity of polyclonal antisera raised against egc proteins. Fifty nanograms of recombinant proteins were employed, antiserum against SEI (first blot), against SE*l*M (second blot), and against SE*l*N (third blot), were tested. PageRuler^TM^ Plus (Thermo Scientific, Vilnius, Lithuania) was added as ladder to identify correct bands (lanes 1 and 7).

In order to determine presumably small amounts of the egc member proteins, we established a polyclonal antibody-based ELISA. Recombinant SEI, SE*l*M, and SE*l*N were produced in our lab and were used to immunize mice (10 µg) and rabbits (30 µg). Antisera were obtained from animals after four rounds of immunization. In order to assess specificities, Western blotting of egc superantigens, as well as of prominent superantigens SEA, SEB, and SEC, was performed. It revealed no cross-reactivity of antisera (Figure 1). 

To further investigate the specificity and cross-reactivity of the polyclonal antibodies that were used as the capture and detecting antibody, 1 µg/mL of SEB, TSST1, as well as various concentrations of endogenous controls (wild-type recombinant SEI, SE*l*M, or SE*l*N) were subjected to the individual ELISA systems. All measurements were normalized by subtraction of corresponding blank values. No antibody showed a significant cross-reactivity in any sandwich ELISA system (Table 2).

**Figure 2:**
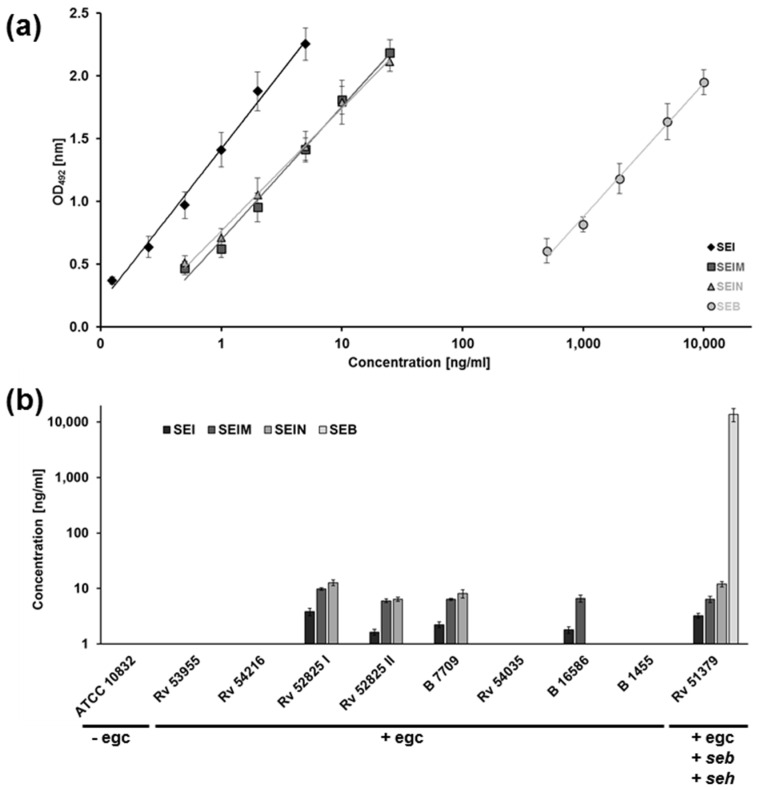
Quantification of proteins of the egc cluster through a polyclonal ELISA system. (**a**) Standard curves of ELISA systems for the detection of SEI, SE*l*M, SE*l*N, and SEB in a lin-log scale. Microtiter plates were coated with the polyclonal murine IgG anti-SE antibody in a dilution of 1:3,000 as the capture antibody. As the detecting antibody, the polyclonal rabbit IgG anti-SE antibody was used in dilution of 1:1,000. The conjugate, a polyclonal horseradish peroxidase-conjugated goat anti-rabbit antibody, was used in a dilution of 1:5,000. Standard curve samples ranged from 0.125 ng/mL to 5 ng/mL for SEI, from 0.5 ng/mL to 25 ng/mL for SE*l*M and SE*l*N, and from 0.5 µg/mL to 10 µg/mL for SEB. The standard curve graphs were obtained by logarithmic regression fit with six (five for SEB) data points made up of the average of duplicate values (SEI *R*^2^ = 0.9925; SE*l*M *R*^2^ = 0.9912; SE*l*N *R*^2^ = 0.9962; SEB *R*^2^ = 0.9948). Error bars indicate the standard error of the mean of five independent experiments; and (**b**) quantification of SEI, SE*l*M, SE*l*N, and SEB in late stationary phase supernatants through polyclonal ELISA system evaluated in (**a**). Samples were 10-fold diluted in 1× PBS containing 2% BSA and 0.1% Tween 20. ATCC 10832 was used as the negative control strain, since it harbors no egc cluster.

For the polyclonal antibody-based ELISA, characteristic standard curve graphs in a lin-log plot with absorbance versus superantigen concentration are shown in Figure 2a. The standard curve graphs generated by the different ELISA systems had comparable slopes—0.53 for SEI, 0.46 for SE*l*M, and 0.43 for SE*l*N, respectively—and gave similar absorbance values for the lowest and highest standard curve samples. The minimum detectable concentration of SEs calculated from the standard curve graph and from positive controls was 0.125 ng/mL for SEI, and 0.5 ng/mL for SE*l*M and for SE*l*N. To verify the polyclonal ELISA setup, we also established a SEB sandwich ELISA based on polyclonal antiserum, and compared its performance with an ELISA system based on a monoclonal SEB antibody (Figure 2a, right panel).

We evaluated the amounts of protein in supernatants of eight egc-positive strains (Figure 2b). As positive and negative controls, we tested two additional strains: the seb-positive isolate Rv51379, and the strain ATCC 10832, which is known to have no prominent superantigens. Accordingly, ELISA for SEI, SE*l*M, and SE*l*N did not show any positive result for ATCC 10832. In contrast, our assay revealed that Rv51379 produced measureable amounts of all three superantigens (SEI/SE*l*M/SE*l*N). Among the eight egc-positive isolates, four supernatants contained enough protein to receive positive results above the detection limit; the remaining four isolates produced no egc proteins or amounts below the detection limit. 

We found that SEI was produced to a lesser extent than SE*l*M and SE*l*N, which displayed the highest concentration in the majority of strains. Overall, amounts of SEI, SE*l*M, and SE*l*N were found to be in a low ng per mL range. In contrast, quantification of SEB in the isolate Rv51379 revealed a 1000-fold higher amount of about 13 µg SEB per mL of culture supernatant. Both SEB ELISA systems displayed almost identical results (Supplementary Materials Table S1). Determination of specificity of each antibody showed no significant cross-reactivity.

### 2.2. Similar SEB and egc Superantigen Concentrations Are Required for Immune Cell Activation

**Figure 3:**
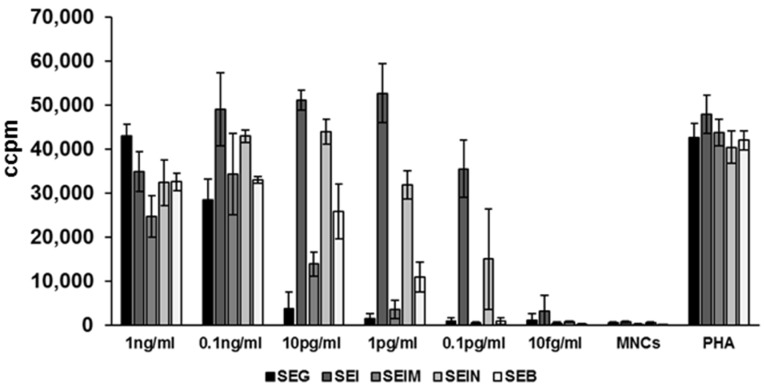
Potentials of recombinant SEG, SEI, SE*l*M, SE*l*N, and SEB to induce proliferation of human peripheral blood mononuclear cells (MNC). Induction of proliferation was quantified by counting incorporated [^3^H] thymidine after incubation for 4 d in a humidified atmosphere (37 °C, 5% CO_2_). Experiments were done with blood from two independent donors. Recombinant wild-type proteins were diluted in RPMI 1640 complete medium. MNCs were adjusted to 1 × 10^6^ cells per mL. Phytohaemagglutinin (PHA) was used as a control.

We tested the distinct potentials of recombinant SEG, SEI, SE*l*M, SE*l*N and, in comparison, SEB, to activate T cells and induce proliferation of human peripheral blood mononuclear cells (Figure 3). We chose a dilution range of 1 ng/mL to 10 fg/mL for each egc protein. All five tested superantigens were able to do so up to a concentration of 0.1 ng/mL. Nevertheless, SEI and SE*l*N continued to induce proliferation until a concentration of 0.1 pg/mL. SEB started to show decreased induction at ≥1 pg/mL. 

**Figure 4:**
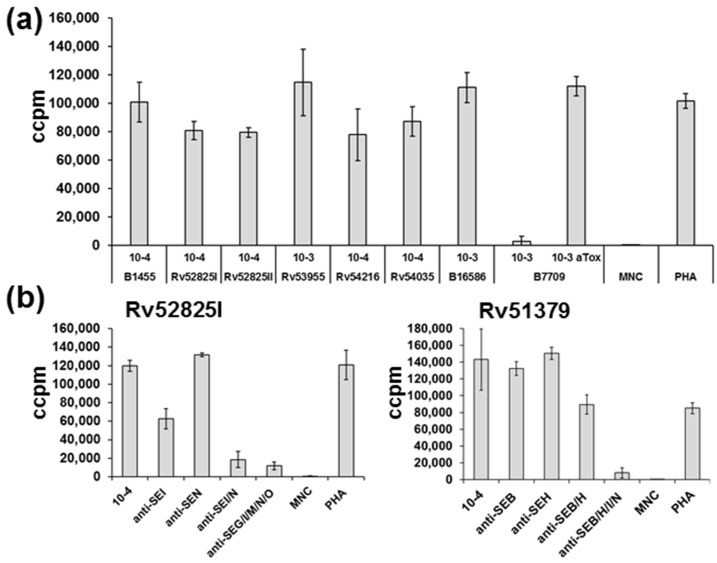
Proliferation of human peripheral blood mononuclear cells (MNC) is stimulated by egc superantigens and can be neutralized by antisera against SEI and SE*l*N. (**a**) Induced proliferation was measured by the counting of incorporated [^3^H] thymidine after incubation for 4 d in a humidified atmosphere (37 °C, 5% CO_2_). Each experiment was performed in triplicate. Supernatants were diluted in RPMI 1640 medium, phytohaemagglutinin (PHA) was used as a control. The supernatant of strain B7709 was additionally incubated with alpha toxin antiserum in a dilution of 1:50 for 1 h at 37 °C (900 rpm); (**b**) Diluted supernatants of Rv52825I or Rv51379 were neutralized with antisera (*x*-axis annotation) in a dilution of 1:50 for 1 h at 37 °C (900 rpm). Different suspensions of egc antisera (left panel), suspensions of anti-SEB/SEH or plus-favored anti-SEI/SElN antisera (right panel), were applied to specific 10^−4^-fold diluted supernatants of tested strains. After incubation, mixes were added to isolated blood cells, and incorporation of [^3^H] thymidine was measured. Experiments were performed as described in (**a**).

Furthermore, we assessed the potential of supernatants of egc strains to induce proliferation of human MNCs (Figure 4a). All supernatants were able to induce proliferation up to a dilution stage of 10^−3^ or 10^−4^. Toxicity due to alpha toxin was only observed in one supernatant (B7709), where alpha toxin concentration was >1 µg/mL [23]. To characterize the impact of distinct egc superantigens, antisera against all members of the egc cluster were applied to the supernatant of the strain Rv52825I, which showed the highest produced amounts of egc proteins tested before (Figure 4b). A supernatant dilution of 10^−4^ resulted in picogram quantities of egc superantigens, a concentration shown to still induce proliferation (see Figure 3).

Strikingly, we found that the dual combination of antisera against SEI and SE*l*N was sufficient to inhibit proliferation. Genomic analysis of isolate Rv51379 revealed two additional prominent superantigen genes (*seb* and *seh*). At the same dilution stage as for the supernatants of egc strains we found that it is essential to neutralize egc superantigens beside SEB and SEH, as well, in order to inhibit the activation of T cells. In line with our previous results, we found that SEI and SE*l*N seem to have the highest potential to induce proliferation. 

### 2.3. Amplification of LPS Toxicity In Vivo Can Be Demonstrated with Members of the egc Operon 

We wanted to know whether our results also reflect the in vivo situation in the host upon infection. At first, we tested SEB and the two egc superantigens, SEI and SE*l*N, in a rabbit model (Table 3). Recombinant wild-type proteins were diluted to 10 µg, and applied subcutaneously to rabbits. Upon 4 h of application, 10 µg LPS was given intravenously. All rabbits died after 24 h, displaying the same high potency of SEB and the egc superantigens.

Thereafter, toxicity of supernatants was explored in this rabbit model (Table 3, lower panel). We picked the isolate Rv52825I, which produced the highest amount of tested egc superantigens. Furthermore, it only expressed very low amounts of a truncated version of alpha toxin, which was also found to be insoluble [23]. Young adult rabbits received 2 mL of supernatant through a subcutaneous injection. According to our quantifications, these loads contained only 7.7 ng of SEI and 25.4 ng of SE*l*N. After 4 h of injection, 100 µg of LPS was applied intravenously. Two rabbits died within 24 h. One rabbit developed severe diarrhoea and showed severe reddened eye mucosa, suffered from heavy breathing, and was highly apathetic. Due to dehydration and acute paralysis of forelegs, the rabbit was euthanized after 72 h. The fourth rabbit displayed fever and moderate reddened eye mucosa after 24 h and suffered from mild diarrhea for 48 h. Altogether, the supernatant of Rv52825I caused a fatal outcome upon application in three out of four rabbits.

To prove our hypothesis about the importance of the egc operon, we explored the distinct impact of SEI and SE*l*N on the outcome of disease in our rabbit model. Before in vivo application, antisera against SEI and SE*l*N had been further characterized and compared to SEB antiserum. Established ELISA systems revealed similar rabbit antibody binding titers for recombinant SEB, SEI, and SE*l*N. After the second vaccination, all tested sera already displayed high titers (>5 × 10^4^), which were defined as dilutions sufficient to give valid detection signals. After the fourth immunization, all sera reached a similar range (>2.5 × 10^5^–10^6^). Thus, all three egc proteins were immunogenic and comparable. Antisera against SEI and SE*l*N were added to the supernatant in a 10-fold dilution and all suspensions were incubated overnight at 37 °C. Four hours after subcutaneous injection, 100 µg of LPS was given intravenously. Strikingly, all four rabbits showed only LPS-associated fever after 24 h, and clinical evidence was negative for 7 days. Hence, it was sufficient to neutralize two out of five superantigens of the egc operon in this supernatant to impede symptoms.

Since both SEI and SE*l*N were shown to play a peculiar role for the pathogenicity of supernatants of egc strains, we finally tested the lethality of very low doses of purified, recombinant SEI and SE*l*N in rabbits (Table 4). In comparison, recombinant SEB was tested in a lower dose, as well. Four hours after subcutaneous injection, 10 µg of LPS were given intravenously. No rabbits died after the application of 1 µg of SEB. In contrast, for both egc superantigens, the application of 10 ng of recombinant proteins was still sufficient to cause lethality after 24 h. Hence, the disastrous effect of very low amounts of egc members could be shown for native and recombinant superantigens. As controls in our rabbit model, we separately tested both high doses of recombinant superantigens (100 µg), and low as well as high doses of LPS (10 µg, 300 µg). The application of superantigens had no effect, while the application of LPS led only to temporary fever. 

## 3. Discussion

The role in disease of prominent staphylococcal enterotoxins, such as SEA, SEB, SEC, or TSST1, has been described extensively [1,24,25]. In contrast, the impact of later-described staphylococcal-like enterotoxins as causative agents in toxin-mediated diseases remained uncertain for a long time. Initially, the importance of SEG and SEI for the toxic shock syndrome and staphylococcal scarlet fever was proposed due to the lack of other superantigens in corresponding isolates [17]. Further involvement of egc in diverse diseases, such as infective endocarditis or food poisoning, have been recently shown [19,26]. 

In a previous study, we found that two thirds of clinical isolates carried the egc in their genomes [23]. Results of several studies agreed that antibodies against egc proteins are lacking in sera of most infected patients even though genomic expression of egc could be detected in the infective strains of staphylococci [27,28]. There could be several explanations for this phenomenon. Egc proteins could be weak antigens. Further, higher mutation rates could impede the recognition of epitopes. Their quantities could not be sufficient to induce an antibody response, or they could be produced at sites in the host unable to induce adaptive immunity. In fact, small amounts might not enter circulation, but act locally. Grumann et al. postulated different phases of interaction as a possible explanation for the lack of an efficient adaptive immune response [29].

In this study we aimed to answer different questions regarding the egc operon. We investigated production rates, immunogenicity, and toxicity of native egc superantigens present in supernatants, and compared the latter with purified, recombinant egc proteins. To identify the proteins, we produced the individual egc proteins by recombinant technology, and obtained the specific antisera against each of the proteins by immunizing rabbits. Sera were shown to be non-cross reactive, and were used as reagents in Western blot and ELISA experiments. Recombinant proteins were used as controls in the identification of the native proteins in bacterial supernatants. At first, sequence analyses revealed no mutational hot spots among members of the egc. Identical amino acids were found in >85% positions, with four members having even a rate >90%.

Transcription of *seg* and *sei* was already found in isolates of a food poisoning outbreak in Japan [21]. Accordingly, transcription was visible in all strains in our study as well, but the increase of mRNA levels of all members of the egc cluster remained low. Measurement was done until the early stationary growth phase, since the peak of transcription is known to be in the exponential phase [29,30]. Overall, measured transcription rates were reproducible, but below a two-fold increase.

Assessment of the proteins SEI, SE*l*M, SE*l*N, and SEB as a control with a newly-developed sandwich ELISA system revealed substantial differences in protein production between SEB and egc proteins. We found more than 10 µg of SEB per mL in the supernatant of the control clinical isolate, whereas members of the egc operon remained at levels around 10 ng per mL. SE*l*N was produced in the highest amounts followed by SE*l*M. Amounts of SEI always remained below 10 ng per mL, which indicated alternative transcription or translation sites. When Omoe and colleagues compared production rates of SEI and SEG in a food poisoning outbreak, they discovered a similar imbalance with SEG showing lower amounts than SEI [21]. Overall, we detected very low amounts of egc proteins throughout all clinical isolates tested. Whether this reflects the situation in the host during colonization or infection cannot be foreseen. Correct assessment of produced amounts would need to include both the blood and organs in animal models. Upon the application of TSST-1 in rabbits we found a rapid compartmentalization of the activated lymphocytes in response to the superantigen toxin within six hours [31]. 

Small amounts of superantigens induce activation of large amounts of T cells leading to cell proliferation and ^3^H thymidine incorporation. [32,33]. Supernatants bearing only members of the egc cluster were shown to display superantigenicity [27]. We analyzed our recombinant egc-associated superantigens, and found that each of the recombinant proteins induced T cell activation in a range of 0.1 pg to 0.1 ng per mL. [29]. When we quantified concentrations of egc toxins in bacterial supernatants, we detected low nanogram amounts. Low levels of egc proteins in supernatants may give an explanation about missing antibody titers in patients: very low doses can already display toxic effects, but are not immunogenic. Lethality of sub-immunogenic doses was shown for neurotoxins [34].

Immunization studies with conventional superantigens, and our unpublished results (MM Eibl and N Model), have revealed microgram doses being as effective to induce a significant antibody response concerning binding and protective antibodies [35,36]. In our present study, immunization with four doses of 30 µg for SEB, SEI, and SE*l*N showed great variation between individual rabbits, but resulted in antibody productions within the same range. Immunization of mice with identical amounts of 10 µg for SEB and egc proteins successfully induced binding antibodies against each of the proteins tested. Taking into consideration the amounts of superantigens in bacterial supernatants detected, it became evident that the amount produced was far below (by log steps) the amounts necessary for immunization. This explains why individuals who had most likely been in contact with these antigens, did not mount an antibody response.

Dauwalder and colleagues found that in comparison to prominent superantigen A, the egc-associated SEG displayed weaker cytokine induction in ex vivo experiments [37]. The overall TNF-α release was higher for SEA at applied nanogram concentrations, whereas IFN-γ was detectable with both toxins. These results indicate, that some members of the egc operon might show a diminished immune response at low doses. We tested microgram amounts of egc and other superantigens. Under these conditions, the response to these antigens was within the same range, not exceeding differences of the response in individuals in an outbred rabbit population. Our results emphasize the importance of SEI and SE*l*N. In a recent report, a staphylococcal strain transduced with an integration vector carrying full length *seg*, *selm*, *selo*, but truncated versions of *sei* and *seln*, led to increased weight loss, but otherwise unaffected mortality in a murine model [38]. 

Superantigenicity of supernatants of isolated *S. aureus* strains could be neutralized by antisera generated with recombinant proteins. We were able to determine the setup of antisera necessary to block T cell activation induced by egc-positive isolates. We tested combinations of antisera against all five egc superantigens and proved protection according to specificity, while SEI and SE*l*N had the highest neutralizing potency. In addition, we could show that blocking of cell proliferation induced by a strain bearing *seb*/*seh* and the egc cluster also depended on the neutralization of SEI/SE*l*N.

Bacterial lipopolysaccharide may be present in low amounts in the circulation. Stress situations, e.g., inflammation, are thought to be important for the translocation of Gram-negative bacteria in the gut and increased endotoxemia [39]. Superantigen toxins were shown to amplify the lethal effect of endotoxins by several log steps in rabbits, which can overcome high amounts of LPS alone. [40,41]. The rabbit model with the application of staphylococcal enterotoxins and subsequent injection of endotoxin is the most sensitive, and is frequently used to define the neutralizing potency of antibodies. In this model, 10 ng of SEI or SE*l*N induced lethality while more than 1 µg of SEB had to be applied for the same effect. Neutralizing antibodies against SEI and SE*l*N in supernatants protected rabbits against a lethal challenge approaching 100%. 

We confirmed that egc proteins are transcribed and secreted at nanogram quantities. This led us to the assumption that infected individuals have been exposed to sub-immunogenic concentrations of the antigen. We were the first to analyze each member in supernatants by inhibiting their function with individual specific antibodies. Here, we further describe that exotoxins of a clinical isolate containing only egc proteins, were lethal in combination with endotoxin for rabbits. We share the opinion that prominent superantigen toxins, such as TSST1, SEB, or SEC, are most important to be included in any vaccination strategies [42]. However, the overall prevalence of the egc cluster, and the fact that many clinical isolates contain nothing but this operon, should be kept in mind. 

## 4. Materials and Methods 

### 4.1. Bacterial Strains, Extraction of RNA, and Isolation of Supernatants

Bacterial strains, growth conditions and primer sequences were described elsewhere [23]. As clinical isolates were anonymous and data of the patients were not accessible, the study was exempt from ethical approval. Protein sequences were aligned using the software Vector NTI Advance (Version 10.3.0, Invitrogen, Carlsbad, CA, USA). The ATCC 10832 strain was purchased from LGC-Standards (Wesel, Germany). For RNA extraction, strains were grown in tryptic soy broth at 37 °C with shaking at 170 revolutions per minute. Pellets from strains were gained at indicated time points and were frozen at −20 °C. RNA was extracted using a High pure RNA isolation kit (Roche, Mannheim, Germany). Concentrations were determined using a Ultrospec 6300 pro spectrophotometer (Amersham Biosciences, Little Chalfont, UK), and quality was checked on RNA agarose gels. RNA was transcribed into cDNA using the SuperScript III kit (Invitrogen) following the manufacturer´s protocol. RNA and cDNA were stored at −20 °C until further usage.

For isolation of superantigens, strains were cultured to stationary phase in 25 mL of tryptic soy broth at 37 °C with shaking at 170 revolutions per minute. In order to gain cell-free supernatants, at indicated time points, cultures were centrifuged at 3,220 × *g* for 5 min, followed by a sterile filtration of supernatants (PALL Acrodisc 25 mm syringe filters with 0.2 µm posidyne membrane). 

All protein samples were stored at −20 °C. Western blots were conducted as previously described [23]. Recombinant proteins were tested in a final concentration of 50 ng. Antisera used as primary antibodies were employed in a 1:40,000 dilution, and secondary antibodies were diluted 1:50,000.

### 4.2. Quantitative Real-Time PCR

General procedure of RT-PCR in our lab was already described in [31]. Concentration of cDNA was checked upon incubation with RNAse H through a spectrophotometer and was adjusted for application, if necessary. All primers (see Supplementary Materials Table S2) were tested to show standard curves with efficiencies between 90% and 110%. Specificity of PCR products was verified visually on agarose gels and by melting curve analysis. Matching samples were always simultaneously amplified on the same plate (MicroAmp, Life Technologies, Carlsbad, CA, USA) using the ABI Prism 7500-FAST with the Kapa SYBR-Fast Super Mix (Peqlab, Erlangen, Germany) and ROX as reference dyes. Due to the work with bacteria, we used two housekeeping genes, *rpoB* and *gyrB*, as internal standards. Differences in cycle thresholds between genes were expressed as a ratio calculated through 2^−∆CP^, where ∆CP is the subtraction between measured crossing points [43]. Fold induction of specific genes was determined from triplicate values normalized against both internal standards. 

### 4.3. Expression and Purification of Proteins

Wild-type recombinant SEB (M11118.1), SEG (BA000017.4), SEI (BA000017.4), SE*l*M (BA000017.4), SE*l*N (EF531605.1), and SE*l*O (BA000017.4) proteins were produced in our lab. *Escherichia coli* strains (One Shot, Invitrogen) transformed with pET vectors expressing superantigen genes (Novagen, Madison, WI, USA), were grown at 28 °C, and expression was induced by arabinose for 24 h. Pellets from SEG and SE*l*N protein-expressing bacteria were resuspended in Tris buffer (pH 7.2), sonicated and centrifuged at 47,000× *g*. Similarly, pellets containing SEI were resuspended in phosphate buffer (pH 6 or pH 7.2). The pellets containing the superantigens SEB or SE*l*M were resuspended in citrate buffer (pH 5). Supernatants were loaded on SP-Sepharose (SEB/SEI/SE*l*M) or Q-Sepharose (SEG/SE*l*N) FF columns (GE Healthcare, Little Chalfont, UK); SEB, SEI, and SE*l*M proteins were eluted with a NaCl gradient, whereas SEG and SE*l*N proteins were part of the flow-through. The peak fractions of SEI/SE*l*M were dialyzed with Tris (pH 7.2/8) and applied to Q-Sepharose FF columns (GE Healthcare). Ammonium sulfate was added to the peak fractions of SEG and the SE*l*N flow-through. Both were applied to Phe-Sepharose FF columns. SEG was eluted using an ammonium sulfate gradient, dialyzed against Tris buffer (pH 8), and applied to a Q-Sepharose FF column. SE*l*N was eluted from an ammonium sulfate-equilibrated column using a pH gradient with citrate buffer pH 8 to pH 5. In the end, all peak fractions were dialyzed against 1× PBS and samples were stored at −20 °C. The pellet of *N*-terminally GST-tagged SE*l*O was resuspended in a PBS buffer with 0.1% Triton X. Supernatant with SE*l*O was applied to GST-Sepharose column. 1 U of factor Xa was added and incubated at 4 °C overnight. Cleaved protein was eluted with 1× PBS. Factor Xa was removed by adding NaCl and benzamidine sepharose. SE*l*O was dialyzed against 1× PBS and stored at −20 °C. 

All used recombinant egc proteins were tested to be LPS free (below the detection limit of the Limulus test) and clean (size exclusion chromatography and host-cell-protein ELISA). For antibody specificity testing, they were compared to prominent staphylococcal superantigens A, B, and C. Further, a protein control gel was stained with Coomassie (Supplementary Materials Figure S1B). 

For the production of antisera, New Zealand White rabbits with a weight between 1.5 and 2 kg were purchased from Charles River Laboratories (Sulzfeld, Germany). Animals were kept in standard facilities with free access to water and food (Ssniff, Soest, Germany), according to the guidelines of the Austrian Ministry for Science and Research. Animal experiments had been approved and controlled by the Veterinary Department of the City of Vienna. For each superantigen, two rabbits and twenty mice were immunized with recombinant wild-type proteins. Antisera were obtained from rabbits after four rounds of immunization with 30 µg for rabbits and 10 µg for mice. Naïve rabbits were challenged with indicated amounts of sterile-filtered supernatants of different strains. Doses were given subcutaneously. Lipopolysaccharide from *Escherichia coli* 0111:B4 (LPS, Sigma, St. Louis, MO, USA; 1 mg/mL; potency: 600,000 EnU/mL) was diluted in 1× PBS, and was given intravenously 4 h after the application of supernatants. Lethality was monitored over a period of seven days. For neutralization of superantigens, antiserum was added in a 1:10 dilution. Samples were incubated at 37 °C overnight.

### 4.4. Enzyme-Linked Immunosorbent Assay

Suitable ELISA conditions for each staphylococcal enterotoxin (SE) and staphylococcal enterotoxin-like (SE*l*) toxin had to be determined. In detail, flat-bottom 96-well plates (Sarstedt, Newton, NC, USA) were coated with 50 μL/well of either the monoclonal antibody or one of the precipitated polyclonal murine IgG antibodies diluted in carbonate buffer pH 9.6. The monoclonal anti-SEB antibody with a concentration of 1 mg/mL was diluted 1:10,000, the polyclonal anti-SEB antibody preparation was used in a final dilution of 1:1,000, and the anti-SEI, anti-SE*l*M, as well as the anti-SE*l*N antibody preparations were used in a final dilution of 1:3000. After 16 to 18 h of incubation at 4 °C, the coated plates were washed four times with washing buffer (PBS pH 7.2, Gibco, Grand Island, NY, USA) containing 0.1% (*v*/*v*) Tween 20 (Merck, Darmstadt, Germany). Thereafter, wells were blocked with 200 µL blocking buffer (PBS containing 2% (*w*/*v*) BSA and 0.1% (*v*/*v*) Tween 20), and incubated for 1 h at room temperature with gentle agitation before they were stored at −20 °C. 

Samples and controls were diluted in blocking buffer, and plates were incubated with 100 μL/well of each standard, sample and control at room temperature for 90 min while receiving gentle agitation. Standard curve samples ranged from 0.125 ng/mL to 5 ng/mL for SEI, and from 0.5 ng/mL to 25 ng/mL for SE*l*M, SE*l*N, and SEB. Bacterial supernatants were diluted in blocking buffer. Plates were washed four times with washing buffer and 100 μL/well of precipitated polyclonal rabbit IgG antibodies were added as detection antibody. The anti-SEB antibody preparation was used in a final dilution of 1:5,000 or 1:3,000 for the monoclonal or polyclonal antibody-based system, respectively. The anti-SEI, anti-SE*l*M, and anti-SE*l*N antibody preparations were used in a final dilution of 1:1,000, and plates were incubated for 1 h under the same conditions as mentioned above. To prepare the conjugate, a polyclonal horseradish peroxidase-conjugated goat anti-rabbit IgG antibody with a concentration of 1 mg/mL was diluted 1:5,000 in blocking buffer. Subsequently, plates were washed as described above, and 50 µL/well of the conjugate were added. Following incubation for 1 h under the same conditions as mentioned before, plates were washed as described above and 100 μL/well of an o-phenylendiamine (Sigma, St. Louis, MO, USA) and H_2_O_2_ substrate were added. Plates were incubated for ten minutes at room temperature in the dark. To stop the colorimetric reaction, 100 μL/well of a 1% (*v*/*v*) sulfuric acid solution were added. The plates were scanned for absorbance at 492 nm wavelength using a spectrophotometer (Tecan, Männedorf, Switzerland).

Antibody titers were determined through an indirect ELISA. Flat-bottomed 96-well plates were coated with 0.5 µg/mL of wild-type recombinant SEB, 0.25 µg/mL of wild-type recombinant SEI, or 1.0 µg/mL of wild-type recombinant SE*l*N in carbonate buffer pH 9.6. Assays were developed as described above; horseradish peroxidase-conjugated goat anti-rabbit IgG-HRP antibodies (GE Healthcare) were added in a 1:20,000 dilution for SEB and SEI, or 1:10,000 for SE*l*N. Titers of antisera were determined and expressed as the inverse of the highest dilution (perofrmed in two-fold dilution steps) for valid detection signals.

### 4.5. Lymphocyte Proliferation Assay

Peripheral blood mononuclear cells (MNC) were isolated from heparinized blood of healthy donors through density gradient centrifugation using Lymphoprep (Axis-Shiels PoC, Oslo, Norway and adjusted to 1 × 10^6^ cells/mL as described elsewhere [31]. In 96-well-flat-bottom tissue culture plates 1 × 10^5^ cells/well were cultured in complete medium (RPMI 1640, Gibco, Grand Island, NY, USA) containing glutamine (GE Healthcare, Little Chalfont, UK), 10% fetal bovine serum (HyClone, Logan, UK), 100 U/mL penicillin, and 100 µg/mL streptomycin (Invitrogen, Carlsbad, CA, USA). Bacterial supernatants diluted in complete medium—with antisera at the indicated concentrations—were incubated for 1 h at 37 °C and continuous movement (900 revolutions per minute). Lectin phytohaemagglutinin (PHA, Sigma, St. Louis, MO, USA) was used in a 1:160 dilution as a positive control. Cells were cultured in a humidified atmosphere (37 °C, 5% CO_2_). On the fourth day, ^3^H-thymidine diluted in complete medium with a final concentration of 0.5 µCi per well (GE Healthcare) was added. After 18 h, plates were frozen at −20 °C overnight before incorporated radioactivity was determined using a MicroBeta Trilux 1450 scintillation counter (Wallac, Turku, Finland). Counts per minute were measured and, based on the automated counter protocols in order to define counting parameters, shown as corrected counts per minute (ccpm).

## Figures and Tables

**Table 1:** Used strains.

Isolates	Staphylococcal Enterotoxins (-Like)
Rv53955	*sei*, *selm*, *seln*, *selo*, *selu*, *selx*
Rv54216	*sei*, *selm*, *seln*, *selo*, *selu*, *selx*
784N-10	*seg*, *sei*, *selm*, *seln*, *selo*
Rv51334	*seg*, *sei*, *selm*, *seln*, *selo*, *selx*
Rv51410	*seg*, *sei*, *selm*, *seln*, *selo*, *selu*
Rv51412	*seg*, *sei*, *selm*, *seln*, *selo*, *selu*
Rv52825 I	*seg*, *sei*, *selm*, *seln*, *selo*, *selx*
Rv52825 II	*seg*, *sei*, *selm*, *seln*, *selo*
B7709	*seg*, *sei*, *selm*, *seln*, *selo*, *selu*, *selx*
B8186	*seg*, *sei*, *selm*, *seln*, *selo*, *selu*
Rv54035	*seg*, *sei*, *selm*, *seln*, *selo*, *selu*, *selx*
B16586	*seg*, *sei*, *selm*, *seln*, *selo*, *selu*
B1455	*seg*, *sei*, *selm*, *seln*, *selo*, *selw*, *selx*
803N-10	*seg*, *sei*, *selm*, *seln*, *selo*, *selu*, *selx*
Rv51379	*seb*, *seg*, *seh*, *sei*, *selm*, *seln*, *selo*, *selu*, *selx*
ATCC 10832	*selx*

**Table 2:** Specificity of the sandwich ELISA system for the detection of SEI, SE*l*M, and SE*l*N.

Sample	Conc.	SEI ^1^	SE*l*M ^1^	SE*l*N ^1^
wt rSEI	5 ng/mL	4.67 ng/mL	0.01 ng/mL	0.02 ng/mL
	1 ng/mL	0.85 ng/mL	0.01 ng/mL	<0.01 ng/mL
wt rSE*l*M	10 ng/mL	0.01 ng/mL	9.62 ng/mL	0.01 ng/mL
	1 ng/mL	0.01 ng/mL	0.74 ng/mL	0.01 ng/mL
wt rSE*l*N	20 ng/mL	0.01 ng/mL	<0.01 ng/mL	17.89 ng/mL
	2 ng/mL	<0.01 ng/mL	<0.01 ng/mL	1.53 ng/mL
wt rSEB	1 ng/mL	0.01 ng/mL	<0.01 ng/mL	0.04 ng/mL
wt rTSST1	1 ng/mL	0.01 ng/mL	0.01 ng/mL	0.05 ng/mL

^1^ means of normalized triplicate measurements.

**Table 3:** Toxicity of supernatants with egc superantigens in a rabbit model.

SE/Strain	Conc.	Vol.	Sera	LPS	No.^1^
rSEB	10 µg	1 mL	-	10 µg	4/4
rSEI	10 µg	1 mL	-	10 µg	2/2
rSE*l*N	10 µg	1 mL	-	10 µg	2/2
Rv52825I	7.7 ng/25.4 ng	2 mL	-	100 µg	3/4
Rv52825I	7.7 ng/25.4 ng	2 mL	anti-SEI/SE*l*N	100 µg	0/4

^1^ Numbers of dead/treated rabbits.

**Table 4:** Toxicity of recombinant superantigens in a rabbit model.

SE	Conc.	Vol.	LPS	No.^1^
rSEB	1 µg	1 mL	10 µg	0/2
rSEI	10 ng	1 mL	10 µg	2/2
rSE*l*N	10 ng	1 mL	10 µg	2/2

^1^ Numbers of dead/treated rabbits.

## Supplementary Materials

The following are available online at www.mdpi.com/2072-6651/8/11/314/s1, Figure S1: (**A**) Relative expression ratios for *seg*, *sei*, *selm*, *seln*, *selo*, and *selu*. Transcription levels of each staphylococcal enterotoxin (SE) gene (G *seg*, I *sei*, M *selm*, N *seln*, O *selo*, U *selu*) were quantified using real-time PCR. Normalization was done using the geometric mean of two reference genes (*rpoB* and *gyrB*). Fold induction is given as the ratio of a distinct egc target gene to the geometric mean of references. Standard deviations are given as standard error of mean for this strain population. Supernatants were taken at the indicated time points. Quantifications were done in triplicate, and results from fifteen strains were combined for each superantigen; (**B**) Control polyacrylamide gel (12%) for Western blot analysis. 50 ng of recombinant proteins were employed, PageRuler^TM^ Plus (Biorad) was added as ladder to identify correct bands (lane 1). Gel was stained with Coomassie Blue for 30 min and destained with an ethanol/acetic acid/water solution, Table S1: Sandwich ELISA system for the detection of SEB, Table S2: RT-PCR Primer Sequences.

## Abbreviations

The following abbreviations are used in this manuscript:

SE	Staphylococcal enterotoxin
SE*l*	Staphylococcal enterotoxin-like
TSS	Toxic shock syndrome
MNC	Peripheral blood mononuclear cells
LPS	Lipopolysaccharide
