# Supplementary material for: Enterotoxin Gene Cluster-Encoded SEI and SElN from Staphylococcus aureus Isolates are Crucial for the Induction of Human Blood Cell Proliferation and Pathogenicity in Rabbits
Source: Toxins (Basel). 2016 Oct 28;8(11):314. doi: 10.3390/toxins8110314 (PMC5127111; doi:10.3390/toxins8110314)
Supplement: Supplementary file 1 [file toxins-08-00314-s001.docx]

Supplementary Materials: Enterotoxin Gene Cluster-Encoded SEI and SE*l*N from *Staphylococcus aureus* Isolates are Crucial for the Induction of Human Blood Cell Proliferation and Pathogenicity in Rabbits

Andreas Roetzer, Corina S. Gruener, Guenter Haller, John Beyerly, Nina Model
and Martha M. Eibl


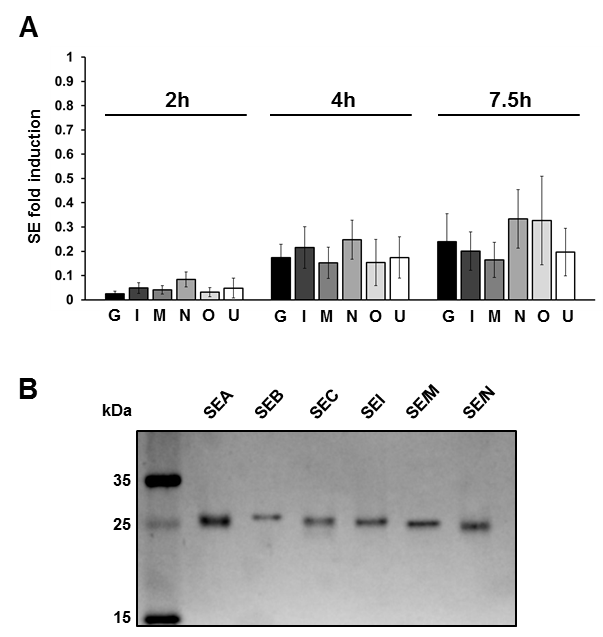


**Figure S1.** (**A**) Relative expression ratios for *seg*, *sei*, *selm*, *seln*, *selo*, and *selu*. Transcription levels of each staphylococcal enterotoxin (SE) gene (G *seg*, I *sei*, M *selm*, N *seln*, O *selo*, U *selu*) were quantified using real-time PCR. Normalization was done using the geometric mean of two reference genes (*rpoB* and *gyrB*). Fold induction is given as the ratio of a distinct egc target gene to the geometric mean of references. Standard deviations are given as standard error of mean for this strain population. Supernatants were taken at the indicated time points. Quantifications were done in triplicate, and results from fifteen strains were combined for each superantigen; (**B**) Control polyacrylamide gel (12%) for Western blot analysis. 50 ng of recombinant proteins were employed, PageRuler^TM^ Plus (Biorad) was added as ladder to identify correct bands (lane 1). Gel was stained with Coomassie Blue for 30 min and destained with an ethanol/acetic acid/water solution.

**Table S1.** Sandwich ELISA system for the detection of SEB.

| **Samples** | | **Conc. (ng/mL)** | |
| --- | --- | --- | --- |
|  |  | **Polyclonal** | **Monoclonal** |
| (+) | wt rec. SEB—10 ng/mL | 8.85 | 10.15 |
| Isolate | Rv51379 | 13.42 × 10^3^ | 13.19 × 10^3^ |
| (−) | wt rec. TSST1—30 ng/mL | 0.04 | 0.01 |

Comparison of the sensitivity of the capture antibody pAb1 (polyclonal) and mAb1715 (monoclonal) used to detect either wild type recombinant SEB, or wild type SEB of the strain Rv51379. No significant cross reactivity with TSST1 was observed. All measurements were normalized by subtraction of blank values. The polyclonal as the monoclonal antibody based system were able to detect samples of defined concentrations (endogenous controls) with a deviation of less than 14%, indicating a sufficient accuracy.

**Table S2.** RT-PCR Primer Sequences.

| **Targets** | **Forward Primer** | **Reverse Primer** | **Anneal** | **Source** |
| --- | --- | --- | --- | --- |
| *seg* | TTACAAAGCAAGACACTGGCTCA | ATATGGAACAAAAGGTACTAGTTC | 58 °C | this study |
| *sei* | GGTACCAATGATTTGATCTCAGAAT | TTTACCAGTGTTATTATGACCATAT | 58 °C | this study |
| *selm* | TCATATCGCAACCGCTGATGATG | ATTTCTCTAAATAATCACCTGCTAA | 58 °C | this study |
| *seln* | GATGAAGAGAAAGTTATAGGCGT | AACTCTGCTCCCACTGAACC | 58 °C | this study |
| *selo* | GTGTAAGAAGTCAAGTGTAGAC | GTACAGGCAGTATCCACTTG | 58 °C | this study |
| *selu* | AATGGCTCTAAAATTGATGGTTC | CCATATTATCCGCTGAAAAATAG | 58 °C | this study |
| *rpoB* | AAGACGGCACTGAAAACACT | ATAACGACCCACGCTTGC | 58 °C | this study |
| *gyrB* | GTAACGGATAACGGACGTGG | ATACTTTGTATCCGCCACCG | 58 °C | this study |

Forward and reverse primer sequences for all genes belonging to the egc operon and the two reference genes are shown together with the specific annealing temperature for the quantitative PCR.
